# MAPK-mediated auxin signal transduction pathways regulate the malic acid secretion under aluminum stress in wheat (*Triticum aestivum* L.)

**DOI:** 10.1038/s41598-017-01803-3

**Published:** 2017-05-09

**Authors:** Xinwei Liu, Yameng Lin, Diqiu Liu, Chengxiao Wang, Zhuqing Zhao, Xiuming Cui, Ying Liu, Ye Yang

**Affiliations:** 1 Yunnan Provincial Key Laboratory of Panax notoginseng, Key Laboratory of Panax notoginseng Resources Sustainable Development and Utilization of State Administration of Traditional Chinese Medicine, Kunming Key Laboratory of Sustainable Development and Utilization of Famous-Region Drug, Faculty of Life Science and Technology, Kunming University of Science and Technology, Kunming, 650500 China; 2 Centre for Microelement Research of Huazhong Agricultural University, Wuhan, 430070 China

## Abstract

An isobaric tags for relative and absolute quantitative (iTRAQ)-based quantitative proteomic approach was used to screen the differentially expressed proteins during control treatment (CK), aluminum (Al) and Al^+^ indole-3-acetic acid (IAA) treatment of wheat lines ET8 (Al-tolerant). Further, the the expression levels of auxin response factor (*ARF*), *Aux*/*IAA*, Mitogen activated protein kinase (MAPK) 2c, and MAPK1a were analyzed. Results showed that 16 proteins were determined to be differentially expressed in response to Al and IAA co-treatment compared with Al alone. Among them, MAPK2c and MAPK1a proteins displayed markedly differential expression during the processes. The expression of *ARF2* was upregulated and *Aux*/*IAA* was downregulated by Al, while both in concentration- and time-dependent manners. Western-blot detection of MAPK2c and MAPK1a indicated that Al upregulated MAPK2c and downregulated MAPK1a in both concentration- and time-dependent manners. Exogenous IAA could promote the expression of MAPK2c, but inhibit the expression of MAPK1a in the presence/absence of Al. These findings indicated that IAA acted as one of the key signaling molecule controls the response mechanism of wheat malic acid efflux to Al stress through the suppression/activation of Aux/IAA and ARFs, and the activity of MAPK2c and MAPK1a were positively or negatively regulated.

## Introduction

A decrease in the accumulation of Al in the root cells is the most effective method of Al resistance. Plants achieve this by chelating rhizosphere-active Al with organic acid that is secreted by the root apex^1^. Organic acid (OA) secretion patterns and transport proteins have been studied extensively^2^. Liu *et al*.^3^ suggested that the Al-induced changes in cytosolic Ca^2+^, pH, K^+^, or ROS trigger the downstream components in the Al signaling pathways. Recently, Li *et al*.^4^ further reported that heterotrimeric G-protein-coordinated transduction of Al-stress signals may be associated with the secretion of OAs from roots of *Arabidopsis* and rye. However, studies detailing the signal regulation mechanism of Al-induced organic acid efflux are still lacking^5^.

Auxin is an important substance for signaling in plants, and it is involved in both growth regulation and heavy metal resistance. For instance, Wang *et al*.^6^ found that *sbIAA1* of sorghum is highly induced under salt and drought stress, and the results further implied that there was cross talk between auxin and abiotic stress signaling pathways. Findings also suggest that Cd stress disturbs auxin homeostasis by affecting auxin level, distribution, metabolism, and transport in Arabidopsis seedling^7^. Application of IAA significantly reduced the Cd accumulation in tissues; hence, increased activity of photosynthesis and antioxidant potential improved the growth performance of *Trigonella* seedlings grown under Cd stress^8^. It has also been demonstrated that auxin negatively regulates aluminum resistance through altering *Al sensitive1* expression and aluminum distribution within *Arabidopsis thaliana* root cells^9^. In response to Al stress, auxin signaling in the root-apex transition zone of *Arabidopsis thaliana* roots is enhanced, which results in auxin-regulated root growth inhibition through a number of ARFs^10^. Previous results involving the topics covered in this paper showed that the efflux of malic acid increased significantly under the co-treatment of IAA and Al, and the content of Al in the root apex decreased; Al-induced malic acid efflux was decreased under the treatments of IAA transport inhibitor N-1-napthyl-phtalamic acid or 2, 3,5-triiodobenzoic acid^11, 12^. Thus, it can be seen that the auxin signaling pathway is involved in the stimulation of malic acid efflux under Al stress. There is a need for more extensive research on this topic.

A number of environmental stress reactions of plants are regulated by MAPK signal transduction pathways. Time-dependent activation of MAPK signaling was seen after exposure to Cu or Cd in leaves of *Arabidopsis thaliana* ^13^. In soybean seedlings, Cd caused induction of genes encoding proteins involved in MAPK cascades, and they possess several regulative motifs associated with the plant response to stress factors and abscisic acid signaling^14^. Moreover, it has also been shown that TMKP1 activity can be modulated by Mn^2+^ ^15^. Reversible protein phosphorylation is related to Al-induced malate/citrate secretion from the wheat/rice bean root apex and the expression of *VuMATE1* and *EcMATE1* ^16–18^. Recently, results from Panda *et al*.^19^ also suggested that MAPK underlies Al-induced DNA damage in root cells of *A. cepa*. However, it is unknown whether MAPKs take part in regulating the Al-induced efflux of wheat malic acid.

MAPK signal transduction pathways play an important role in the auxin signal transduction system. Lee *et al*.^20^ demonstrated that MPK12 is both a physiological substrate of *INDOLE-3-BUTYRIC ACID-RESPONSE5* and a novel negative regulator of auxin signaling in *Arabidopsis*. Marqués-Bueno *et al*.^21^ proposed that protein kinase CK2 functions in the regulation of auxin-signaling pathways, particularly in auxin transport. Researchers also identified MAPK6 involved in *Arabidopsis* post-embryogenic root development through auxin up-regulation and cell division plane orientation and mitotic microtubule (PPB and phragmoplast) organization^22^. Highly specific and transient expression of a MAP Kinase determines auxin-induced leaf venation patterns in *Arabidopsis* ^23^. Works also found that *T. atroviride* regulates root architecture and increases MAPK6 activity, depending on auxin signaling^24^.

Proteomics is a powerful tool for the analysis of interaction of protein to protein. To date, many researchers have been proposed to different proteome-based techniques, including the newly emergent mass spectrometry method using isobaric tags for relative and absolute quantification (iTRAQ)^25, 26^. iTRAQ-based mass spectrometry is a technique of multiplexed protein quantitative mass spectrometry using amine-reactive isobaric tagging reagents. The advantage of this technique is that it enables quantitation of multiple samples simultaneously but requires only a small amount of sample.

In this study, we used iTRAQ-based proteomics to assess proteome changes and identify proteins that were differentially expressed in wheat roots in response to Al treatment or Al and IAA co-treatment. We examined the effects of IAA and protein kinase inhibitors on wheat malic acid secretion, the expression of the auxin response gene and MAPK under aluminum stress. Moreover, the MAPK pathway-mediated auxin signal transduction system and Al-induced malic acid efflux will be proved further.

## Results

### iTRAQ analysis and identification of differentially expressed proteins

**Figure 1:**
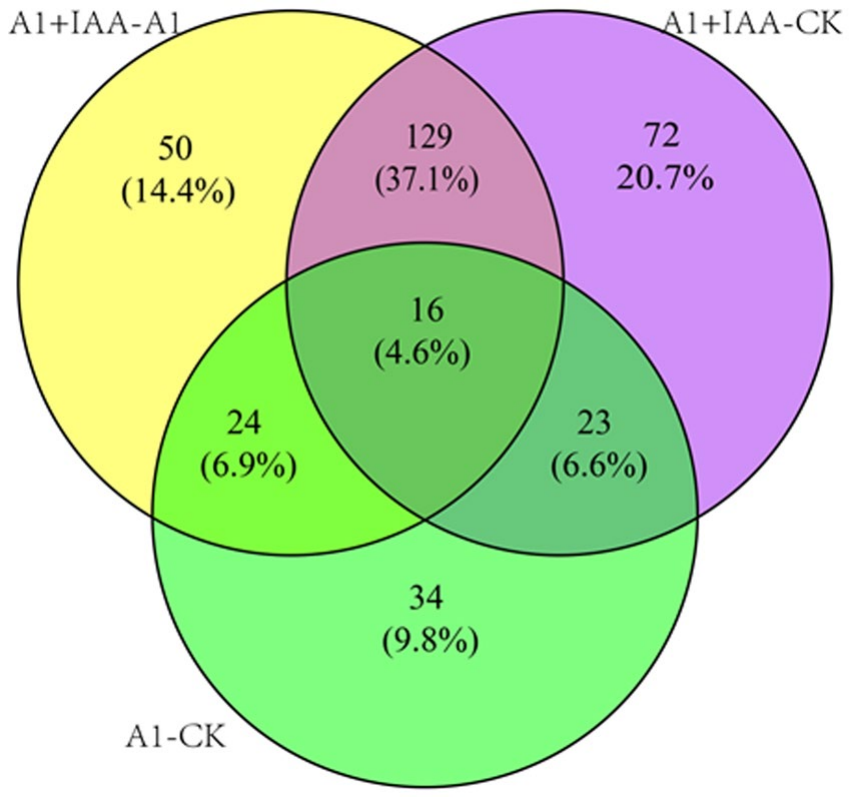
Venn diagram of the relationship of intersection of differentially expressed proteins responsive to Al and IAA co-treatment compared with Al alone in roots of wheat.

Total proteins were extracted from the wheat root (untreated control (CK), Al, IAA + Al); two biological replicates were used for each condition, and were subjected to iTRAQ labeling and LC-MS/MS analysis. In total, 348 proteins were assembled, of which 16 (4.6%) differentially expressed proteins (DEPs) displayed (Fig. 1).

### Differentially expressed proteins with Al alone-treatment or Al and IAA co-treatment

The reporter peaks of the iTRAQ tags in the MS/MS spectra were used for quantification. A total of 16 proteins were determined to be differentially expression during Al and IAA stress compared with Al alone-treatment based on having a fold change >1.5 or <0.67 (*p* < 0.05) (Supplementary Table 1). Among them, 7/16 was known and 9/16 was uncharacterized. These known proteins were involved in stress response, metabolic regulation and anti-oxidative damage. The majority of the differentially expression proteins were stress response including three members (Q8S9H0, Q5BU11, A9RAB2), three metabolic regulation proteins (W5GG94, W5E0K5, A0A077RTX0), and one anti-oxidative damage proteins (Q8GTC0).

### Effects of IAA on the efflux of malic acid

**Figure 2:**
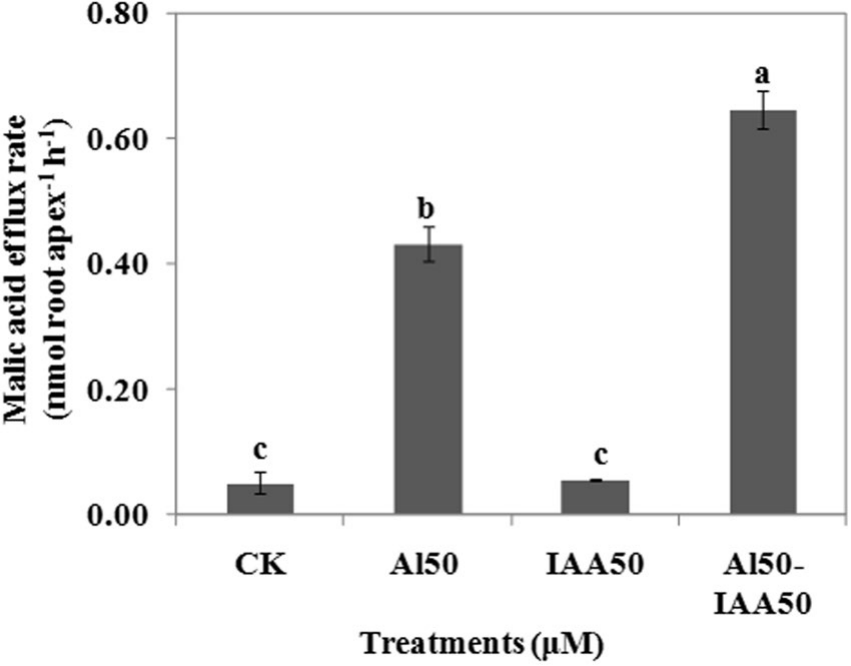
Effects of IAA on malic acid efflux from 4-day-old wheat seedlings. Seedlings were exposed to 0.5 mM CaCl_2_ solution (pH 4.5) containing other chemicals: 0 μM AlCl_3_ (CK), 50 μM AlCl_3_, 50 μM IAA, 50 μM AlCl_3_ and 50 μM IAA for 24 h. Data are means ± SD (n = 3). Small letter differences in the same figure mean significant difference at *P* < 0.05.

The efflux of malic acid under 50 μM IAA additions was equivalent to that of the control, which indicated that IAA alone could not induce the efflux of malic acid. While, co-treatment with 50 μM IAA and 50 μM Al induced increase of the malic acid efflux rate by 49.6% (Fig. 2). These results indicate that IAA signaling participates in the Al-induced efflux of malic acid.

### IAA alleviates the inhibition effects of Phenylarsine oxide on the efflux of malic acid

**Figure 3:**
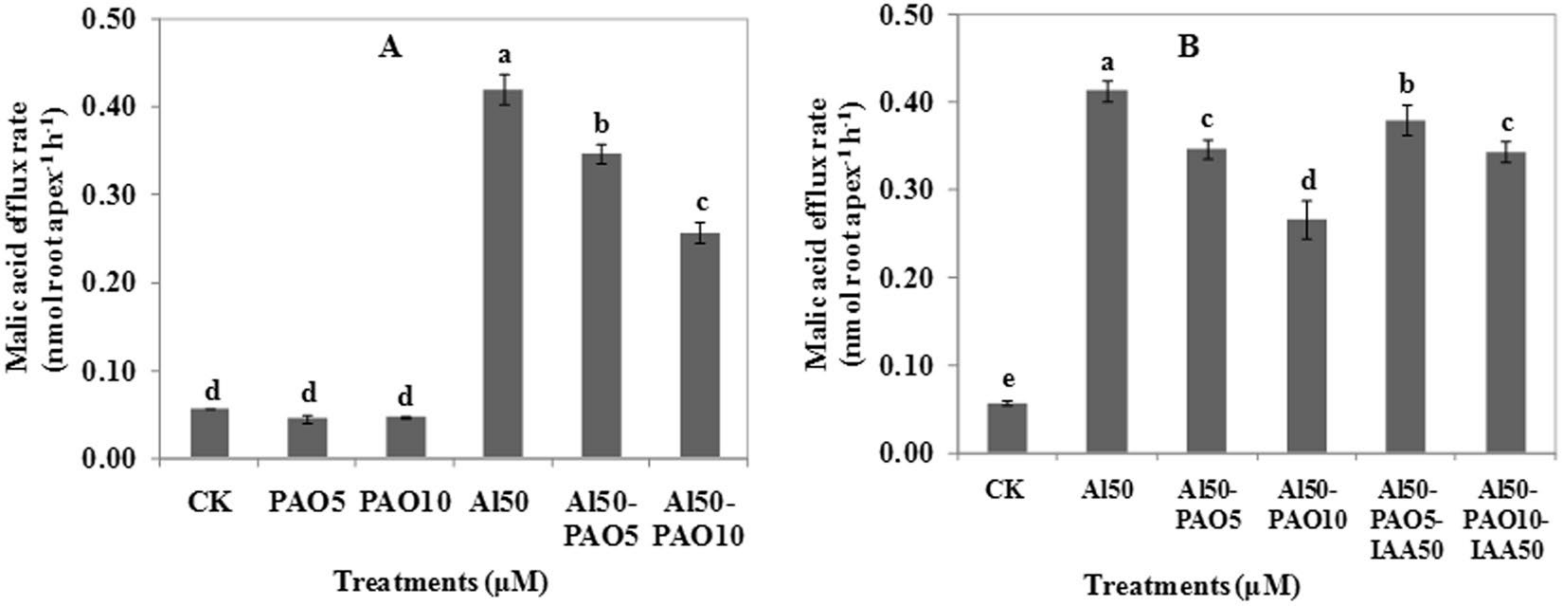
Effects of protein kinase inhibitor PAO and IAA on malic acid efflux from 4-day-old wheat seedlings. Seedlings were exposed to 0.5 mM CaCl_2_ solution (pH 4.5) containing other chemicals: (**A**) 0 μM AlCl_3_ (CK), 5 (10) μM PAO, 50 μM AlCl_3_, 50 μM AlCl_3_ and 5 (or 10) μM PAO for 24 h; (**B**) 0 μM AlCl_3_ (CK), 50 μM AlCl_3_, 50 μM AlCl_3_ and 5 (or 10) μM PAO, 50 μM AlCl_3_ and 50 μM IAA and 5 (or 10) μM PAO for 24 h. Data are means ± SD (n = 3). Small letter differences in the same figure mean significant difference at *P* < 0.05.

Phenylarsine oxide (PAO) is a specific inhibitor of protein tytorsine phosphatases (PTPases). The efflux of malic acid under the treatment of 5 or 10 μM PAO was not different from that of the control, which indicated that PAO could not induce the efflux of malic acid. Compared with 50 μM Al treatment alone, treatment of 5 μM or 10 μM PAO and 50 μM Al co-treatment decreased the malic acid efflux rate by 17.5% or 25.9%, respectively (Fig. 3A). The effect of PAO and IAA on Al-induced malic acid efflux is shown in Fig. 3B. Compared with the co-treatment of Al and PAO, the application of IAA (50 μM Al, 5 μM or 10 μM PAO, and 50 μM IAA) significantly induced the efflux of malic acid. The addition of 50 μM IAA to the solutions containing 5 μM or 10 μM PAO induced malic acid efflux rate was 0.38 or 0.34 nmol root apex^−1^ h^−1^, respectively which was 1.12 and 1.27 times higher than the co-treatment of PAO and Al, respectively. These results preliminary concluded that IAA signaling acted on protein kinase to activate the Al-induced efflux of malic acid.

### Effects of Al on the expression of *ARF2* gene

**Figure 4:**
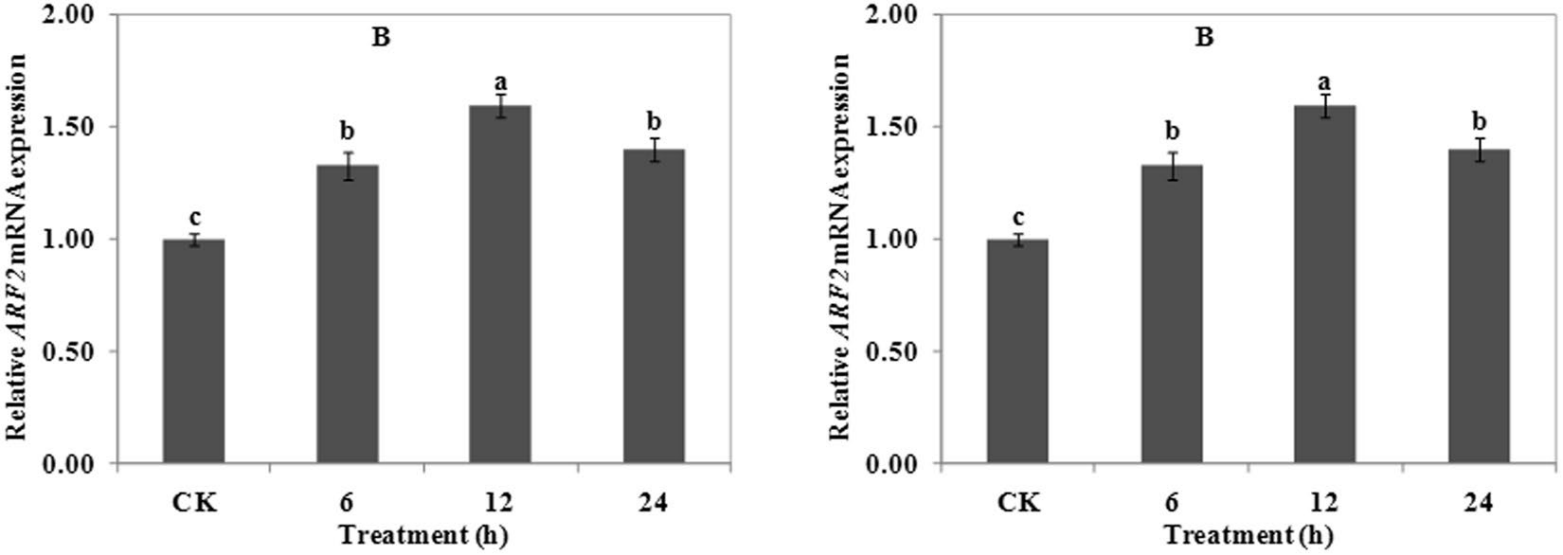
Expression of *ARF2* in wheat. Real time quantitative RT-PCR was used to assess *ARF2* expression in root apexes (0–20 mm) of wheat lines ET8. Four-day-old wheat seedlings were exposed to 0.5 mM CaCl_2_ solution (pH 4.5) containing 0 (CK), 25, 50 or 100 μM AlCl_3_ for 24 h (**A**). Four-day-old wheat seedlings were exposed to 0.5 mM CaCl_2_ solution (pH 4.5) containing 0 or 50 μM AlCl_3_ for 0 (CK), 6, 12 and 24 h (**B**). Data are means ± SD (n = 3). Small letter differences in the same figure mean significant difference at *P* < 0.05.

Analysis of the root apex by real-time quantitative RT-PCR verified *ARF2* expression (Fig. 4). The expression of *ARF2* was upregulated with increasing concentration of Al, and expression was significantly higher than that of CK. Among the four treatments, 50 µΜ Al treatments elicited the highest expression of *ARF2* (Fig. 4A). Al-induced expression of *ARF2* also depended on induction time, and the highest expression level occurred after 12 h Al exposure (Fig. 4B). The above results demonstrated that Al treatment could induce the expression of *ARF2* in both a concentration- and time- dependent manner.

### Effects of Al on the expression of *Aux*/*IAA* gene

**Figure 5:**
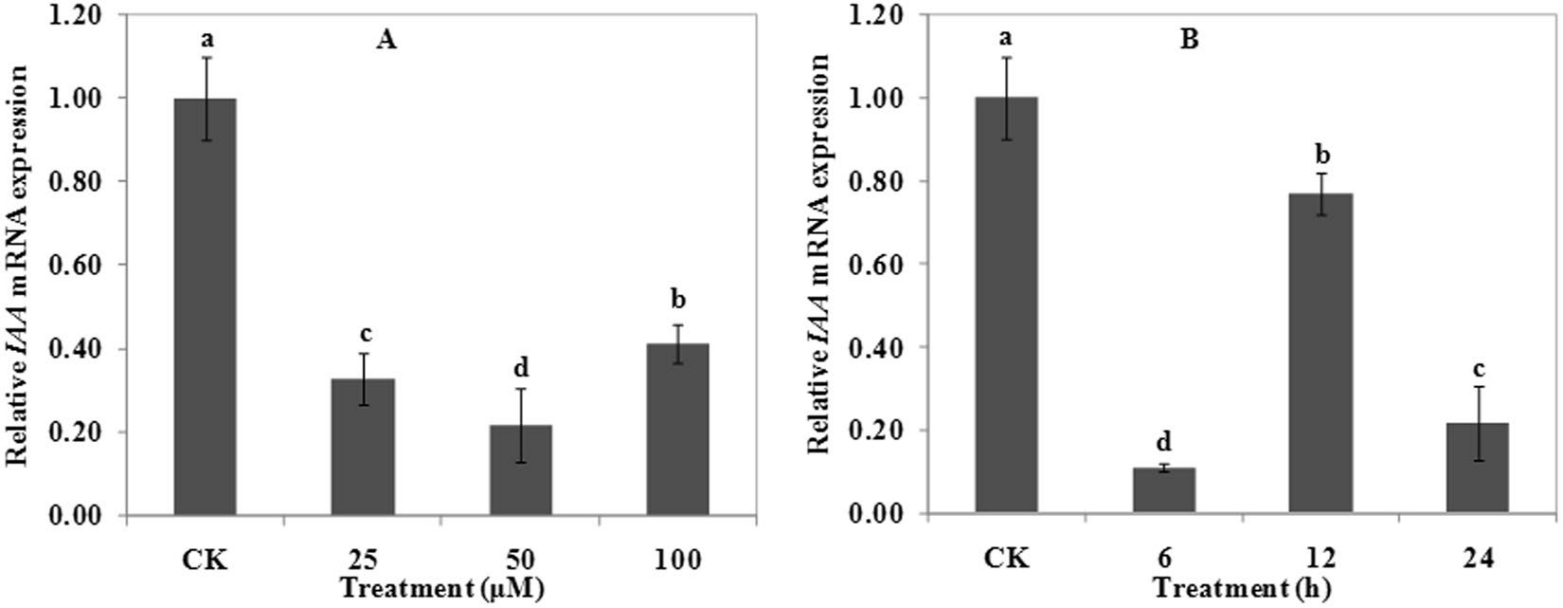
Expression of gene *IAA* in wheat. Real time quantitative RT-PCR was used to assess gene *IAA* expression in root apexes (0–20 mm) of wheat lines ET8. Four-day-old wheat seedlings were exposed to 0.5 mM CaCl_2_ solution (pH 4.5) containing 0 (CK), 25, 50 or 100 μM AlCl_3_ for 24 h (**A**). Four-day-old wheat seedlings were exposed to 0.5 mM CaCl_2_ solution (pH 4.5) containing 0 or 50 μM AlCl_3_ for 0 (CK), 6, 12 and 24 h (**B**). Data are means ± SD (n = 3). Small letter differences in the same figure mean significant difference at *P* < 0.05.

Compared with CK, Al treatment significantly inhibited the expression of *Aux*/*IAA*. The negative regulation effect of 50 μM Al treatment was the most significant among the three concentrations of Al treatments (Fig. 5A). Inhibition effects of *Aux*/*IAA* expression were also observed under the time course treatment (6, 12, and 24 h at 50 μM Al). The lowest expression of *Aux*/*IAA* occurred after 6 h treatment (Fig. 5B). Results demonstrated that Al treatment inhibited the expression of *Aux*/*IAA*.

### Effects of Al on the expression of MAPK2c

**Figure 6:**
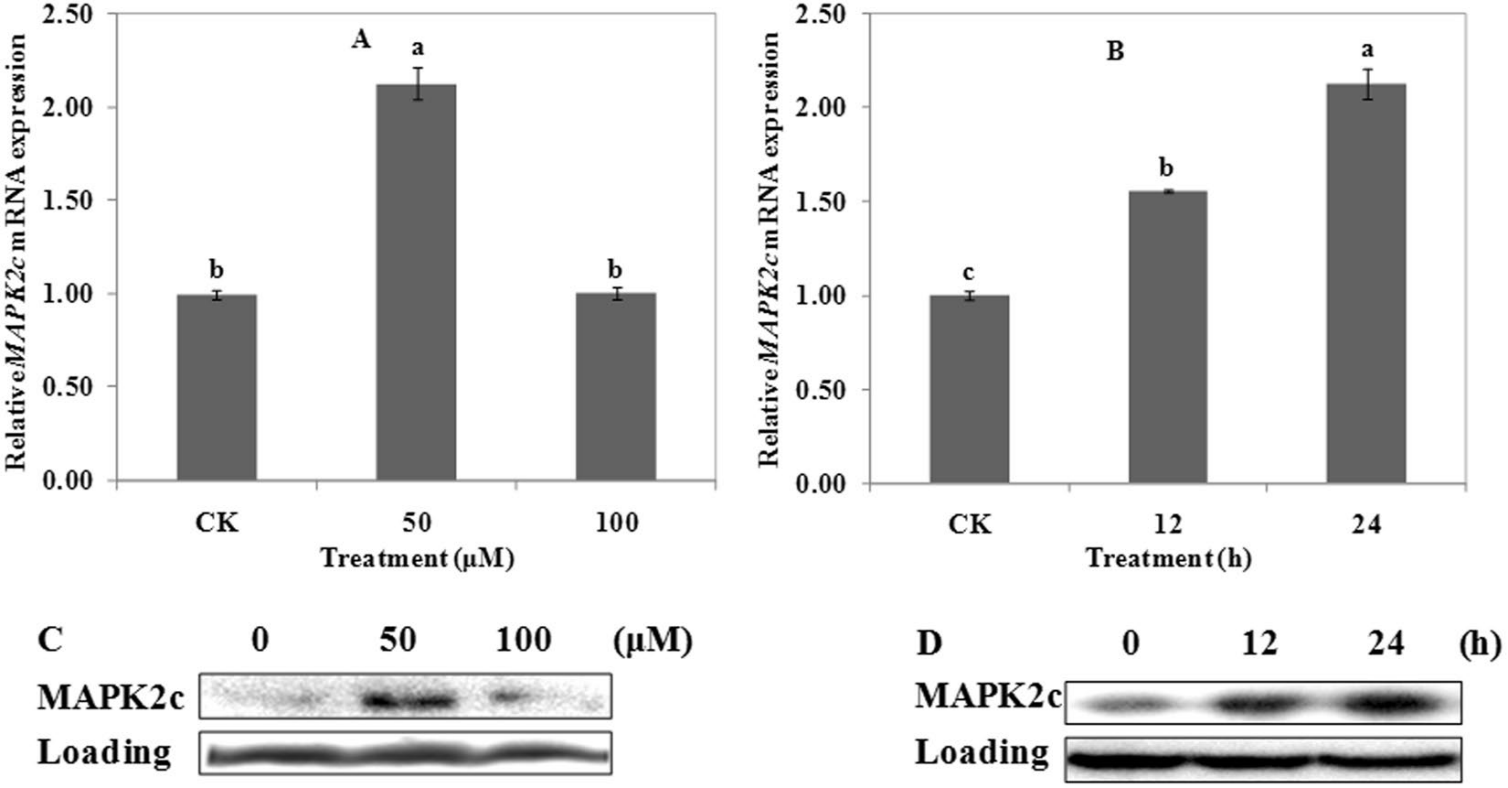
Expression and western blot detection of MAPK2c in wheat. Real time quantitative RT-PCR and western blot were used to assess MAPK2c expression in root apexes (0–20 mm) of wheat lines ET8. Four-day-old wheat seedlings were exposed to 0.5 mM CaCl_2_ solution (pH 4.5) containing 0 (CK), 50 or 100 μM AlCl_3_ for 24 h (**A** and **C**). Four-day-old wheat seedlings were exposed to 0.5 mM CaCl_2_ solution (pH 4.5) containing 0 or 50 μM AlCl_3_ for 0 (CK), 12 and 24 h (**B** and **D**). Data are means ± SD (n = 3). Small letter differences in the same figure mean significant difference at *P* < 0.05.

Expression of gene *MAPK2c* under Al stress was shown in Fig. 6. Compared with CK, 50 μM Al significantly increased the expression of *MAPK2c*. However, there was not any difference between treatment of CK and 100 μM Al (Fig. 6A). The expression level of *MAPK2c* increased with increasing time-course under the treatment of 50 μM Al. The significant expression level occurred after 24 h of treatment (Fig. 6B).

Treatment with 50 μM Al induced the expression of MAPK2c protein; there was less difference between treatment of CK and 100 μM Al (Fig. 6C). 50 μM Al treated for 24 h induced the highest expression of MAPK2c protein (Fig. 6D). Results were consistent with those presented in Fig. 6A and B. These results indicated that MAPK2c protein responded to Al positively.

### Effects of Al on the expression of MAPK1a

**Figure 7:**
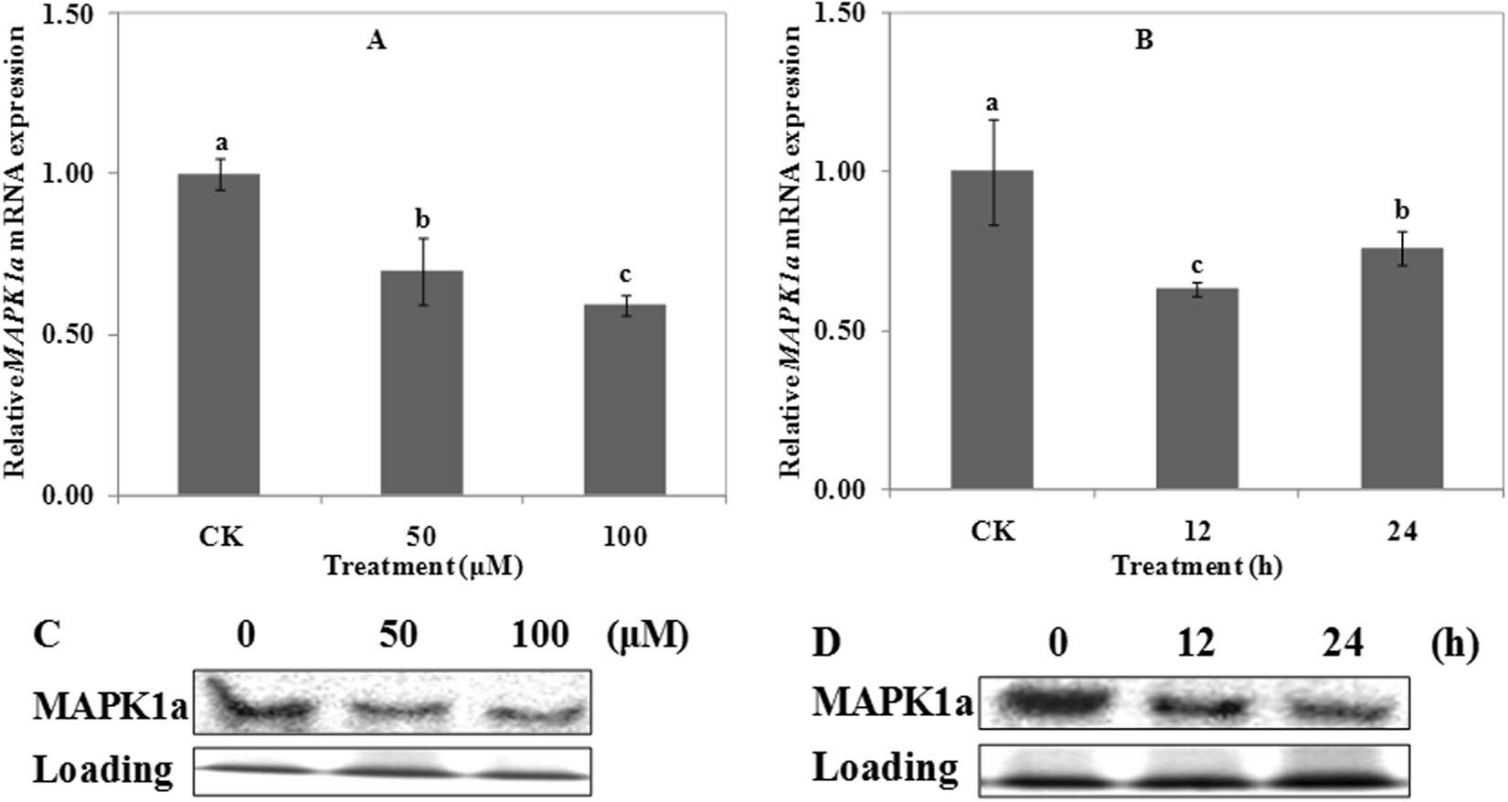
Expression and western blot detection of MAPK1a in wheat. Real time quantitative RT-PCR and western blot were used to assess MAPK1a expression in root apexes (0–20 mm) of wheat lines ET8. Four-day-old wheat seedlings were exposed to 0.5 mM CaCl_2_ solution (pH 4.5) containing 0 (CK), 50 or 100 μM AlCl_3_ for 24 h (**A** and **C**). Four-day-old wheat seedlings were exposed to 0.5 mM CaCl_2_ solution (pH 4.5) containing 0 or 50 μM AlCl_3_ for 0 (CK), 12 and 24 h (**B** and **D**). Data are means ± SD (n = 3). Small letter differences in the same figure mean significant difference at *P* < 0.05.

Expression of *MAPK1a* gene is shown in Fig. 7. Compared with CK, Al treatment inhibited the expression of *MAPK1a* (Fig. 7A). After 50 μM Al treatment for 12 h or 24 h, the expression level of *MAPK1a*s were all lower than CK. The lowest expression level occurred after 12 h of treatment (Fig. 7B).

Compared with CK, treatment of 50 μM and 100 μM Al significantly inhibited the expression of MAPK1a protein, and there was less difference between them (Fig. 7C). The decreased expression of MAPK1a protein became time-dependent after 50 μM Al treatment (Fig. 7D). Expression of MAPK1a protein was in consistent with the results of *MAPK1a* gene. The above results indicated that the expression of MAPK1a protein negatively responded to Al treatment.

### Effects of IAA on the expression of MAPK2c

**Figure 8:**
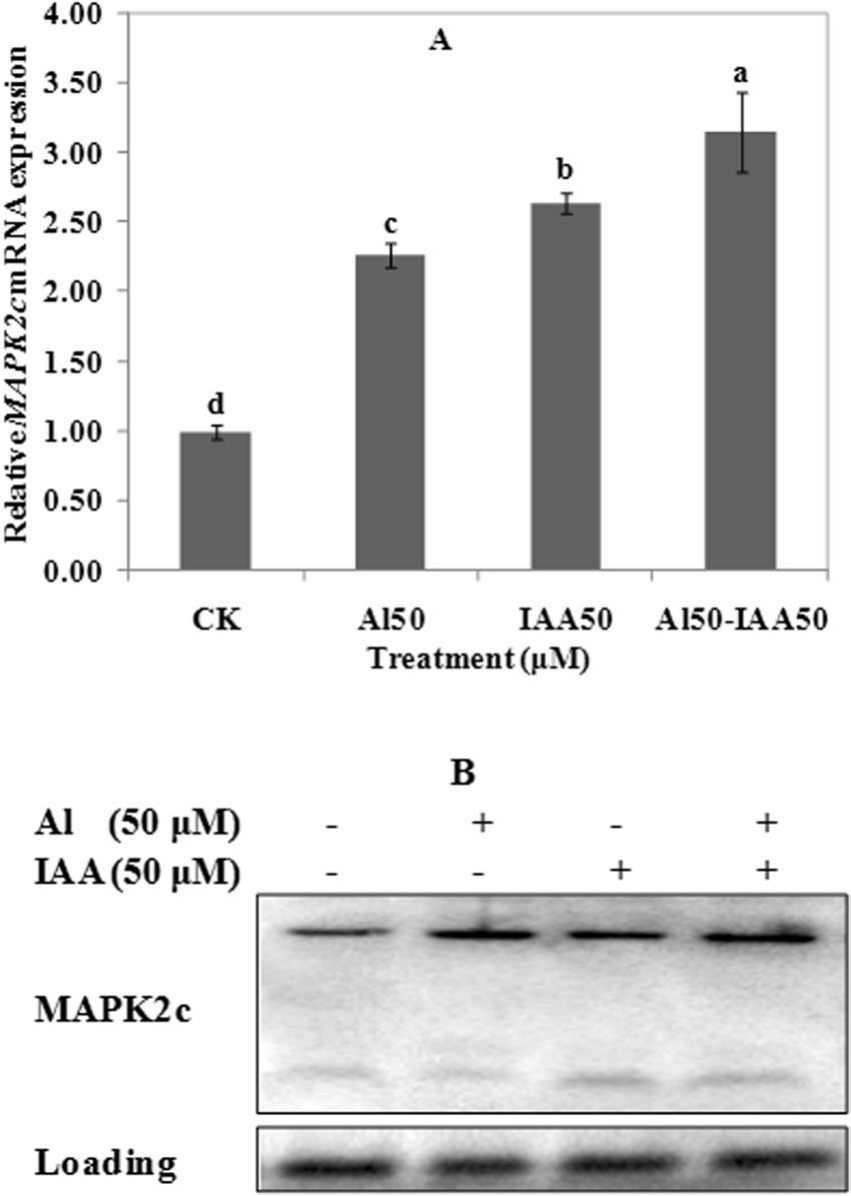
Expression of MAPK2c in wheat, RNA (**A**) and protein (**B**). Real time quantitative RT-PCR and western blot was used to assess MAPK2c gene and protein expression in root apexes (0–20 mm) of wheat lines ET8. Seedlings were exposed to 0.5 mM CaCl_2_ solution (pH 4.5) containing other chemicals: 0 μM AlCl_3_ (CK), 50 μM AlCl_3_, 50 μM IAA, 50 μM AlCl_3_ and 50 μM IAA for 24 h. Data are means ± SD (n = 3). Small letter differences in the same figure mean significant difference at *P* < 0.05.

Compared with 50 μM Al alone treatment, 50 μM IAA or co-treatment of 50 μM IAA and 50 μM Al significantly induced *MAPK2c* expression (Fig. 8A). The expression of MAPK2c protein was also induced by IAA alone treatment or the co-treatment of IAA and Al (Fig. 8B). The expression quantity of MAPK2c protein treated by IAA+Al was significantly higher than Al or IAA alone treatment. Results indicated that IAA positively regulated the expression of MAPK2c protein, and there was a positive cooperative effect with Al.

### Effects of IAA on the expression of MAPK1a

**Figure 9:**
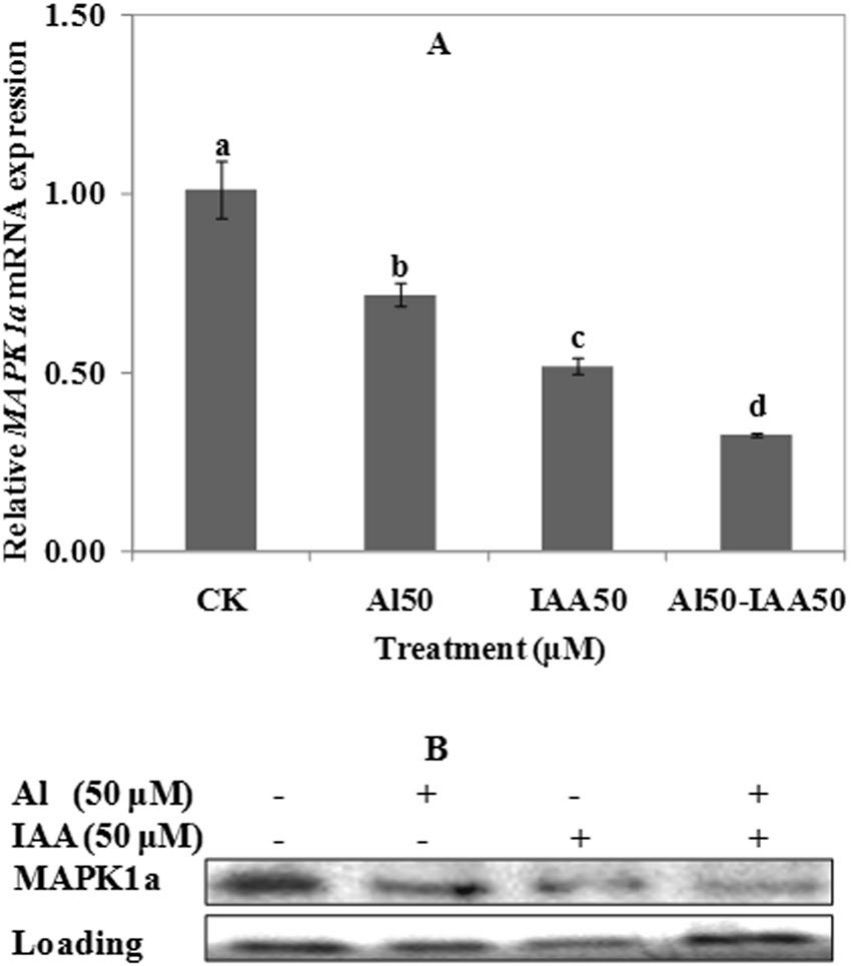
Expression of MAPK1a in wheat, RNA (**A**) and protein (**B**). Real time quantitative RT-PCR and western blot was used to assess MAPK1a gene and protein expression in root apexes (0–20 mm) of wheat lines ET8. Seedlings were exposed to 0.5 mM CaCl_2_ solution (pH 4.5) containing other chemicals: 0 μM AlCl_3_ (CK), 50 μM AlCl_3_, 50 μM IAA, 50 μM AlCl_3_ and 50 μM IAA for 24 h. Data are means ± SD (n = 3). Small letter differences in the same figure mean significant difference at *P* < 0.05.

Effect of IAA on the expression of MAPK1a is shown in Fig. 9. Compared with CK, 50 μM Al treatment, 50 μM IAA treatment or the co-treatment of 50 μM IAA and 50 μM Al all inhibited the expression of *MAPK1a* (Fig. 9A). Compared with CK, expression of protein MAPK1a was inhibited by the treatment of Al or IAA alone, or co-treatment of IAA and Al (Fig. 9B). Results demonstrated that IAA negatively regulated the expression of MAPK1a protein, and there was a negative cooperative effect with Al.

### Effects of PAO on the expression of MAPK2c and MAPK1a

**Figure 10:**
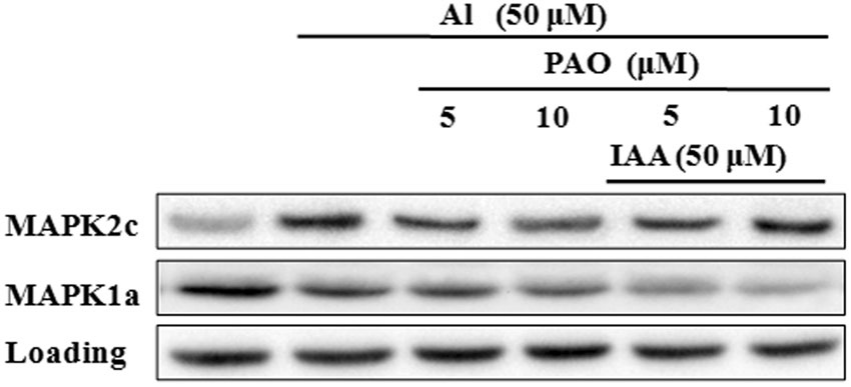
Expression of MAPK2c and MAPK1a in wheat. Western blot was used to assess MAPK2c and MAPK1a protein expression in root apexes (0–20 mm) of wheat lines ET8. Seedlings were exposed to 0.5 mM CaCl_2_ solution (pH 4.5) containing other chemicals: 0 μM AlCl_3_ (CK), 50 μM AlCl_3_, 50 μM AlCl_3_ and 5 (or 10) μM PAO, 50 μM AlCl_3_ and 50 μM IAA and 5 (or 10) μM PAO for 24 h.

The effect of PAO on the expression of MAPK2c and MAPK1a is shown in Fig. 10. Compared with 50 μM Al treatment alone, the co-treatment of 5 (or 10) μM PAO and 50 μM Al significantly inhibited the expression of MAPK2c. Increasing concentration of PAO resulted in decreasing expression quantity of MAPK2c. Expression quantity of MAPK2c protein treated by IAA+PAO+Al was higher than PAO+Al treatment. Compared with 50 μM Al treatment alone, the co-treatment of 5 (or 10) μM PAO and 50 μM Al significantly inhibited the expression of MAPK1a protein. Increasing concentration of PAO resulted in decreasing expression quantity of MAPK1a protein. The expression quantity of protein MAPK1a treated by IAA+PAO+Al were lower than PAO+Al. However, the expression quantities of MAPK1a of all the treatments were lower than CK. The results above indicated that PAO negatively regulated the expression of Al activated MAPK2c, and IAA was antagonism with PAO; PAO positively regulated the expression of Al inhibited MAPK1a, and IAA was cooperative with PAO.

## Discussion

Wheat plants have booted up complex Al-responsive signaling and metabolic processes at the cellular, organ and even whole-plant levels to deal with Al stress. Chelating of rhizosphere-active aluminum by malic acid secreted through an anion channel on the root tip is an important mechanism for Al resistance of wheat^2^. Our preliminary studies shown that efflux of malic acid of wheat cannot stimulate by exogenous IAA alone, but enhanced by the co-treatment of Al, and effectively reduced the content of aluminum in root tip cells^11, 12^. It manifested that there must be some downstream components regulated by IAA signal pathway. However, the exact mechanism of how IAA signaling participates in the Al-induced efflux of malic acid is still unknown. Therefore, an iTRAQ-based quantitative proteomic analysis was conducted of roots with Al or Al and IAA co-treatment, in order to obtain the key effectors during the efflux of malic acid. In the study, a total of 16 differently expression proteins were identified among 7041 identified proteins, among the identified differently expression proteins, MAPKs (MAPK1a, Q5BU11; MAPK2c, A9RAB2) exhibited marked changes in responded to Al and IAA co-treatments compared with Al alone-treated (Supplementary Table 2; Fig. 1).

MAPK cascades are important for plant signal transduction, as they are involved in signaling of hormones, growth factors, microbes, or damage-associated molecular patterns, and they convert extracellular stimuli into intracellular responses while amplifying the transmitting signal^27^. It has been shown that protein kinase participates in Al-stress resistance. Al-responsive citrate excretion of *Eucalyptus camaldulensis* was affected by both protein kinase (K-252a and staurosporine) and phosphatase inhibitors (calyculin A and cyclosporinA)^16^. However, it is not yet known whether or not MAPKs participate in malic acid secretion of wheat regulated by the IAA signaling pathway under Al stress.

In the present study, PAO treatment alone significantly inhibited the root elongation (Supplementary Fig. 1), but affected the efflux of malic acid slightly (Fig. 3A). This showed that PAO alone treatment cannot affect the efflux of malic acid, but caused the interruption of normal metabolic of phosphorylation. For example, Fujimoto *et al*.^28^ found that PAO significantly inhibited internalization of cellulose synthase 3 from the plasma membrane. Al induced efflux of malic acid was inhibited by PAO (Fig. 3A), but could be interrupted by the addition of exogenous IAA (Fig. 3B). Thus, we preliminary concluded that protein kinases may participate in the Al activated malic acid efflux achieved by the IAA signaling pathway. This study focused on genes that responded to Al stress on the IAA signaling pathway.

When confronted with excessive heavy metals, plants could dynamically and differentially regulate the transcription of auxin-related genes to adjust the location and effective accumulation of auxin within the plant for better adaptation and survival under the adverse environment^29^. In recent years, IAA participated in the Al resistance of plants has been reported frequently. Kobayashi *et al*.^30^ identified that IAA could induce *AtALMT1* transcription. It was also found that the modulation of PIN2-based auxin transport, IAA efflux, and cell wall acidification, lines over expressing *OsPIN2* alleviate Al-induced cell rigidity in rice root apex^31^. The expressions of auxin transporter-like protein and auxin efflux carrier component are significantly higher in Al-stressed *alfalfa* roots than in the control, while the expression of auxin conjugates hydrolase is significantly lower^32^. IAA-amino acid hydrolase *ILR1-like 4-like* expression is detected only in boron and Al co-treated roots^33^.

The auxin signaling pathway is mainly composed of auxin signal receptor recognition, signal transduction, and response of downstream genes. The current study found that Aux/IAA proteins and auxin response factors (ARFs) are the two key proteins in the process of auxin signal transduction^34, 35^. Characteristics of the auxin signaling process are as follows: (1) under the condition of low auxin concentration, Aux/IAA and other transcription repressors restrain the transcription activation of ARF and further prevent the transcription of downstream response genes; (2) under the condition of high auxin concentration, auxin combines with Aux/IAA protein and ARFs, which form a complex, then Aux/IAA is degraded by 26 s proteasome, ARF is released from the restraint of Aux/IAA, and the transcription of the downstream gene is activated. Zeng *et al*.^36^ showed that ARFs in soy beans are responsive to Al stress. Research conducted by Kobayashi *et al*.^30^ found that expression of *ARF19* in *Arabidopsis* could not be regulated by Al treatment, but was changed by exogenous IAA. These results illustrated that Al or IAA may have a significant impact on the expression of *ARF*.

The present study found that Al treatment positively regulated the expression of *ARF2* (Fig. 4) and negatively regulated the expression of *Aux*/*IAA* (Fig. 5, Supplementary Table 3), which may be the result of Al-induced accumulation of IAA in the wheat root^11^. Yuan *et al*.^37^ found that Cd inhibits root meristem growth by stabilized AXR3/IAA17 protein to repress auxin signalling mediated process of Arabidopsis. Although changes of IAA content and relative gene were different, the IAA signal pathway participate in response to heavy metal stress was confirmed. We concluded that by regulating the expression of *ARF2* and *Aux*/*IAA*, the expression of downstream genes could be regulated. Changes of *Aux*/*IAA* and *ARF2* expression under the treatment of Al confirmed that the IAA signaling pathway participated in Al resistance of wheat.

Numerous studies have shown that MAPKs are involved in stress resistance, active regulation of channel proteins, signal transduction, and even Al stress resistance. Results have suggested that a short treatment with Al could induce the activity of certain antioxidant enzymes in tobacco cells, and that this response is mediated by a MAPK signal transduction pathway^38^. Salicylic acid attenuates aluminum toxicity by affecting a signaling pathway linked to protein phosphorylation^39^. These results suggest that Al-responsive organic acid efflux through an anion channel involves a protein phosphorylation/dephosphorylation process. This study proved further protein kinase involvement in the efflux of malic acid that is regulated by IAA signaling under Al stress (Fig. 3).

Regarding multiple plant species, phylogenetic analysis suggested that plant MAPKs can be grouped into five groups (A, B, C, D and E)^40^. Group B contains TaMAPK1 and TaMAPK2, which is known to be associated with variety environmental and hormonal responses. These two MAPKs have been well studied and appeared to be involved in environmental stress responses^41–43^. So, these two kinds MAPKs were also studied in present study. Results showed that Al stress significantly affected the expression of MAPK1a and MAPK2c, but other MAPKs (MAPK1f, MAPK2b, MAPKflrs) were influenced less (Supplementary Figs 2–4). Thus, MAPK1a and MAPK2c were further studied under Al or other treatments.

It was found that exogenous Al or IAA treatment significantly induced the expression of MAPK2c (Fig. 8), but it inhibited the expression of MAPK1a (Fig. 9). PAO also significantly inhibited the expression of MAPK2c and MAPK1a (Fig. 10). So, results obtained in present study could strongly demonstrate that MAPKs participated in malic acid efflux under Al stress.

## Conclusions

In summary, this study showed that ARF2, Aux/IAA, MAPK2c and MAPK1a displayed markedly differential expression response to Al treatment. Al upregulated gene *ARF2* and downregulated *Aux*/*IAA*, upregulated MAPK2c and downregulated MAPK1a all in concentration- and time-dependent manners. The inhibition effects of PAO on Al induced efflux of malic acid could be mitigated by the application of IAA. Exogenous IAA upregulated the expression of MAPK2c, but downregulated the expression of MAPK1a in the presence/absence of Al. PAO both negatively regulated the expression of MAPK2c and MAPK1a under Al stress, and IAA was negatively/positively cooperative with PAO, respectively. Our study concluded that IAA is a key signaling molecule that triggers an increase of malic acid against Al toxicity in wheat.

## Materials and Methods

### Plant materials and culture conditions

Seeds of wheat (*Triticum aestivum* L.) lines ET8 (Al-tolerant) were immersed in 1% (v/v) sodium hypochlorite for 15 min for the purpose of surface-sterilization, rinsed several times with deionized water, and then soaked for about 12 h before germination on a layer of moistened filter paper at 25 °C for 24 h in darkness. The germinated seeds were then transferred onto a net made of cotton floating on 0.5 mM CaCl_2_ (pH 4.5) in a 2 L plastic container; the solution was renewed daily. After 4 days, some seedlings of uniform length were selected for experiments. The solution was adjusted to pH 4.5 with 0.2 M HCl and renewed every other day. All experiments were done in an environmentally controlled growth room with a 24 h cycle of 14 h at 25 °C in light/10 h at 22 °C in darkness, a photon flux density of 150 μmol photon m^−2^ s^−1^ (photosynthetic active radiation) at the plant-canopy level, and a relative air humidity of 70%.

### Treatments

This study adopted 7 types of treatments, all of which contained 0.5 mM CaCl_2_ treatment as the control treatment (CK). All treatments initiated by exposing the seedlings for 24 h to 250 mL 0.5 mM CaCl_2_ (pH 4.5). Additional chemicals differed between the treatments. Ten 4-day-old seedlings were selected for the treatment. Each experiment was conducted three times.

Treatment 1: CK; 50 μM Al (AlCl_3_·6H_2_O, *Alfa Aesar*, Lancaster); 50 μM IAA (Sigma, USA); 50 μM Al and 50 μM IAA.

Treatment 2: CK; 5 μM PAO (Sigma, USA); 10 μM PAO; 50 μM Al; 50 μM Al and 5 μM PAO; 50 μM Al and 10 μM PAO.

Treatment 3: CK; 50 μM Al; 50 μM Al and 5 μM PAO; 50 μM Al and 10 μM PAO; 50 μM Al, 5 μM PAO, and 50 μM IAA; 50 μM Al, 10 μM PAO, and 50 μM IAA.

Treatment 4: CK, 25, 50, or 100 μM Al.

Treatment 5: 50 μM Al treated for 0 (CK), 6, 12, and 24 h, respectively.

Treatment 6: CK, 50, or 100 μM Al.

Treatment 7: 50 μM Al treated for 0 (CK), 12 and 24 h, respectively.

At sampling time, solutions of treatment 1, 2 and 3 were collected for the determination of malic acid content. Roots of treatments 4–7 were rinsed briefly with deionized water, and root apexes (0–20 mm) of each seedling were then excised with a razor. The excised roots were stored in liquid nitrogen, homogenized with a pestle, and placed in a microcentrifuge tube to be used for gene or protein expression analysis of *ARF2*, *Aux*/*IAA*, *MAPK2c*, *MAPK1a*, *MAPKfls*, *MAPK1f*, and *MAPK2b*.

### Protein extraction, digestion, iTRAQ labeling and LC-MS/MS analysis

For iTRAQ analysis, samples were first ground to powder with liquid nitrogen. To extract proteins from the samples, the powder was dissolved with 200 μL TEAB dissolution buffer, ultrasonic wave for 15 min, then centrifugation at 12000 r/min for 20 min, the supernatant subsided by adding 4-fold volume cold acetone containing 10 mM DTT for about 2 h, followed by centrifugation at 2000 rpm/min for 20 min at 4 °C, the precipitate was collected and mixed with 800 μL cold acetone at 56 °C to break proteins’ disulfide bonds. Again centrifugation at 12000 r/min for 20 min at 4 °C, the precipitate was collected and dissolved with 100 μL TEAB dissolution buffer.

A total of 100 μg of protein from each sample was dissolved in a dissolution buffer, and then diluted with 500 μL 50 mM NH_4_HCO_3_. After reduced and alkylated, 2 μg trypsin was added and then incubated overnight at 37 °C for protein digestion. After protein digestion, equal volume of 0.1% FA was added for acidize. Peptides were purified on Strata–X C18 pillar which was first activated with methanol and then balanced by adding 1 mL 0.1% FA for three times, washed with 0.1% FA + 5% ACN twice, and eluted with 1 mL 0.1% FA + 80% ACN. Eluted peptides were dried with vacuum concentration meter. The dried peptides power was redissolved with 20 μL 0.5 M TEAB for peptides labeling. There sets of iTRAQ samples were used for the three biological replicates.

Samples were labeled with iTRAQ Reagent-8 plex Multiplex Kit (AB Sciex U.K. Limited) according to the manufacturer’s instructions. All of the labeled samples were mixed with equal amount. Then the labeled samples were fractionated using high-performance liquid chromatography (HPLC) system (Thermo DINOEX Ultimate 3000 BioRS) using a Durashell C18 (5 μm, 100 Å, 4.6 × 250 mm). At last, 12 fractions were collected.

LC-ESI-MS/MS analysis was performed on an AB SCIEX nanoLC-MS/MS (Triple TOF 5600 plus) system. Samples were chromatographed using a 90 min gradient from 2–30% (buffer A 0.1% (v/v) formic acid, 5% (v/v) acetonitrile, buffer B 0.1% (v/v) formic acid, 95% (v/v) acetonitrile) after direct injection onto a 20 μm PicoFrit emitter (New Objective) packed to 12 cm with Magic C18 AQ 3 µm 120 Å stationary phase. MS1 spectra were collected in the range 350–1,500 m/z for 250 ms. The 20 most intense precursors with charge state 2–5 were selected for fragmentation, and MS2 spectra were collected in the range 50–2,000 m/z for 100 ms; precursor ions were excluded from reselection for 15 s.

### Determination of malic acid content

The solution was first passed through a cation-exchange column (16 mm × 140 mm) filled with 5 g of Amberlite IR-120B (H^+^ form) resin (Muromachi Chemical, Tokyo), and then through an anion-exchange column filled with 2 g of Dowex 1 × 8 resin (100–200 mesh, formate form) in a cold room. Malic acid retained on the anion-exchange resin was eluted with 10 mL 2 M HCl, and the eluent was concentrated in a rotary evaporator at 40 °C. The residue was then dissolved in deionized water and measured with enzymic methods^1^. The sample (1.35 mL) was incubated with 1.5 mL of buffer (0.5 M Gly, 0.4 M hydrazine, pH 9.0), and 0.1 mL of 40 mM NAD (Ames Co., USA). The reaction mixture was incubated for 30–60 min to obtain a stable A340 reading before the addition of 5 μL of malate dehydrogenase (Sigma, USA). The increase in A340 due to the production of NADH was measured with a spectrophotometer (Hitachi, U-1800, Japan) and is directly proportional to the amount of malic acid in the sample.

### Gene information and primer designer

Full-length of *MAPK*, *TaARF2*, and *Aux*/*IAA*-encoding genes were inquired from the web of NCBI (Genebank: ARF2, AY902381.1; Aux/IAA. AJ575098.1; MAPK1a, AY881102.1; MAPK2c, DQ322667.1; MAPK1f, DQ444321.1; MAPK2b, DQ322666.1; MAPKflrs, JX646783). Primers were designed by primer 5.0 software (Table 1). All primers were synthesized by Beijing Genomics Institute.

**Table 1:** Full-length of *MAPK*, *TaARF2* and *IAA*-encoding genes.

Target genes	Primer sequence
*MAPK1a*	Forward: 5-GACTGCAAACTGAAAATTTGT-3
*MAPK1a*	Reverse: 5-ACATAATTCAGGGGCTCTATA-3
*MAPK2c*	Forward: 5-GATTGTAAGCTCAAAATATGTG-3
*MAPK2c*	Reverse: 5-ACATAGTTCAGGTGCTCGGTA-3
*MAPKflrs*	Forward: 5-GCAAACTGTGACCTAAAA-3
*MAPKflrs*	Reverse: 5-ACAGAAGCTCTGGTGCC-3
*MAPK2b*	Forward: 5-GATTGTAAGCTCAAAATATG-3
*MAPK2b*	Reverse: 5-ACATAGCTCAGGTGCTCGGTA-3
*MAPK1f*	Forward: 5-GACTGCAAACTGAAAATTTGTG-3
*MAPK1f*	Reverse: 5-ACATAATTCAGGGGCTCTGTA-3
*18 s RNA*	Forward: 5-GGGATAGATCATTGCAATTGT-3
*18 s RNA*	Reverse: 5-TCCGAACACTTCACCGGACCA-3
*TaARF2*	Forward: 5-TGACGCAGTCTCCTCCCACAC-3
*TaARF2*	Reverse: 5-GAAAATGAAGGCGTCCCCAGC-3
*IAA1 Protein*	Forward: 5-TTCTTGCAGTGTGCTATGGTTTC-3
*IAA1 Protein*	Reverse: 5-TAGGGAACATCACATAACACCAATC-3

### RNA isolation and real-time RT-PCR with SYBR green detection

For total RNA isolation, the samples were thawed at room temperature and homogenized in 1 mL of Trizol reagent (Invitrogen, Life science technology, USA), and an RNA prep pure kit was used according to the manufacturer’s protocol. Deoxyribonuclease I (Invitrogen) treatment was performed to remove DNA contamination. RNA concentrations were measured using a NanoDrop ND-1000 Spectrometer (NanoDrop Technologies, Wilmington, USA). The quality and quantity of the RNA were assessed at 260/280 A, and all samples showed absorbency ratios ranging from 1.8 to 2.0. For the RT-PCR, 4 μg of total RNA was mixed with 1 μL of Oligo (dT)_18_ primer (Thermo, Thermoscientific technology, USA), 200 U of RevertAid M-MuL Virus RT (Thermo) in the presence of 20 U of RiboLock RNase inhibitor (Thermo). Oligonucleotide primers for *TaARF2*, *Aux*/*IAA*, and *18 S* were designed using Primer Express Software (Applied Biosystems, Foster City, CA). After RT, 20 ng of cDNA from the same cDNA batch was subjected to RT-PCR to amplify all genes in triplicate in a total reaction volume of 15 μL using Roche SYBR Green Master mix (Roche), and the required amount of forward and reverse primers. Reactions were conducted on an Lightcycler 96 (Roche, Roche Life Science, CH) using the following cycling conditions: pre-incubation at 95 °C for 10 min, 3-step amplication at 95 °C for 10 sec, 60 °C for 30 sec, and 72 °C for 1 min. Anon-template reaction served as the negative control for each experiment. Melting curve analysis of the products as well as amplicon size verification on a 2% agarose gel confirmed the specificity of the PCR. The raw expression level for each gene was calculated using the same external standard curve made with a mixture of cDNA samples.

### Western blot analysis

The samples were re-suspended with lysate lysis buffer (Sangon, Beijing) and lysed on ice for 3–4 h. After 12,000 × *g* centrifugation for 20 min, the protein content of the supernatant was determined by a BCA protein assay kit (Sigma Co., St. Louis, MO, USA). Equivalent amounts of total proteins were separated on 10% dodecyl sulfate, sodium salt (SDS)-Polyacrylamide gel electrophoresis (PAGE) gels and blotted onto a PVDF (polyvinylidene fluoride) membrane. Blots were probed with MAPK-specific antibodies according to the supplier’s guidelines (Beijing Genomics Institute).

### Statistical analysis

Data were pooled and subjected to one-way analysis of variance (ANOVA) followed by Turkey tests. P ≤ 0.05 was set as the level of statistical significance. DPS v7.05 and OrigenPro7.5 software were used for computation, data analysis, and graphics.

## Electronic supplementary material

Supplementary information

Supplementary Dataset 1

Supplementary Dataset 2
