# Supplementary material for: MAPK-mediated auxin signal transduction pathways regulate the malic acid secretion under aluminum stress in wheat (Triticum aestivum L.)
Source: Sci Rep. 2017 May 9;7:1620. doi: 10.1038/s41598-017-01803-3 (PMC5431644; doi:10.1038/s41598-017-01803-3)
Supplement: Supplementary file 1 — Supplementary information [file 41598_2017_1803_MOESM1_ESM.doc]

**SUPPLEMENTARY INFORMATION TO**

**MAPK-mediated auxin signal transduction pathways regulate the malic acid secretion under aluminum stress in wheat (*Triticum aestivum* L.)**

Xinwei Liu^1,2^, Yameng Lin^1,2^, Diqiu Liu^1^, Chengxiao Wang^1^, Zhuqing Zhao^2^, Xiuming Cui^1^, Ying Liu^1*^, Ye Yang^1*^

***Corresponding author**

**Supplementary Figure 1** **Effects of protein kinase inhibitors PAO and IAA on relative root elongation rate of 4-day-old wheat seedlings.** Seedlings were exposed to 0.5 mM CaCl_2_ solution (pH 4.5) containing other chemicals: (A) 0 μM AlCl_3_ (CK), 50 μM AlCl_3_, 5 (10) μM PAO, 50 μM AlCl_3_ and 5 (or 10) μM PAO for 24 h. Data are mean ± SD (n=3). Small letter differences in the same figure indicate a significant difference at *P*<0.05.

**Supplementary Figure 2** **Western blot detection of MAPK1f in wheat.** Four-day-old wheat seedlings were exposed to 0.5 mM CaCl_2_ solution (pH 4.5) containing 0, 50 or 100 μM AlCl_3_ for 24 h (A). Four-day-old wheat seedlings were exposed to 0.5 mM CaCl_2_ solution (pH 4.5) containing 0 or 50 μM AlCl_3_ for 0, 12 and 24 h (B).

**Supplementary Figure 3** **Western blot detection of MAPKflrs in wheat.** Four-day-old wheat seedlings were exposed to 0.5 mM CaCl_2_ solution (pH 4.5) containing 0, 50 or 100 μM AlCl_3_ for 24 h (A). Four-day-old wheat seedlings were exposed to 0.5 mM CaCl_2_ solution (pH 4.5) containing 0 or 50 μM AlCl_3_ for 0, 12 and 24 h (B).

**Supplementary Figure 4** **Western blot detection of MAPK2b in wheat.** Four-day-old wheat seedlings were exposed to 0.5 mM CaCl_2_ solution (pH 4.5) containing 0, 50 or 100 μM AlCl_3_ for 24 h (A). Four-day-old wheat seedlings were exposed to 0.5 mM CaCl_2_ solution (pH 4.5) containing 0 or 50 μM AlCl_3_ for 0, 12 and 24 h (B).

**Supplementary Figure 5** The uncropped image for the expression detection of MAPK2c in wheat. Four-day-old wheat seedlings were exposed to 0.5 mM CaCl_2_ solution (pH 4.5) containing 0 (CK), 50 or 100 μM AlCl_3_ for 24 h . Four-day-old wheat seedlings were exposed to 0.5 mM CaCl_2_ solution (pH 4.5) containing 0 or 50 μM AlCl_3_ for 0 (CK), 12 and 24 h.

**Supplementary Figure 6** The uncropped image for the expression detection of MAPK1a in wheat. Four-day-old wheat seedlings were exposed to 0.5 mM CaCl_2_ solution (pH 4.5) containing 0 (CK), 50 or 100 μM AlCl_3_ for 24 h. Four-day-old wheat seedlings were exposed to 0.5 mM CaCl_2_ solution (pH 4.5) containing 0 or 50 μM AlCl_3_ for 0 (CK), 12 and 24 h.

**Supplementary Figure 7** The uncropped image for the expression detection of MAPK2c in wheat. Western blot was used to assess MAPK2c expression in root apexes (0-20 mm) of wheat lines ET8. Seedlings were exposed to 0.5 mM CaCl_2_ solution (pH 4.5) containing other chemicals: 0 μM AlCl_3_ (CK), 50 μM AlCl_3_, 50 μM IAA, 50 μM AlCl_3_ and 50 μM IAA for 24 h.

**Supplementary Figure 8** The uncropped image for the expression detection of MAPK1a in wheat. Western blot was used to assess MAPK2c expression in root apexes (0-20 mm) of wheat lines ET8. Seedlings were exposed to 0.5 mM CaCl_2_ solution (pH 4.5) containing other chemicals: 0 μM AlCl_3_ (CK), 50 μM AlCl_3_, 50 μM IAA, 50 μM AlCl_3_ and 50 μM IAA for 24 h.

**Supplementary Figure 9** The uncropped image for the expression detection of MAPK2c in wheat. Western blot was used to assess MAPK2c protein expression in root apexes (0-20 mm) of wheat lines ET8. Seedlings were exposed to 0.5 mM CaCl_2_ solution (pH 4.5) containing other chemicals: 0 μM AlCl_3_ (CK), 50 μM AlCl_3_, 50 μM AlCl_3_ and 5 (or 10) μM PAO, 50 μM AlCl_3_ and 50 μM IAA and 5 (or 10) μM PAO for 24 h.

**Supplementary Figure 10** The uncropped image for the expression detection of MAPK1a in wheat. Western blot was used to assess MAPK1a protein expression in root apexes (0-20 mm) of wheat lines ET8. Seedlings were exposed to 0.5 mM CaCl_2_ solution (pH 4.5) containing other chemicals: 0 μM AlCl_3_ (CK), 50 μM AlCl_3_, 50 μM AlCl_3_ and 5 (or 10) μM PAO, 50 μM AlCl_3_ and 50 μM IAA and 5 (or 10) μM PAO for 24 h.

**Supplementary Table 3** Expression of gene *Aux*/*IAA* in wheat. Real time quantitative RT-PCR was used to assess gene *IAA* expression in root apexes (0-20 mm) of wheat lines ET8.

|  | Concentration (μM) | | | |  | Time (h) | | | |
| --- | --- | --- | --- | --- | --- | --- | --- | --- | --- |
|  | 0 | 25 | 50 | 100 |  | 0 | 6 | 12 | 24 |
| TaIAA3-A | 1.04±0.16 | 0.80±0.04 | 0.34±0.09 | 0.42±0.09 |  | 1.04±0.23 | 0.25±0.03 | 0.86±0.13 | 0.33±0.07 |
| TaIAA3-B | 1.00±0.04 | 0.91±0.02 | 0.59±0.10 | 0.70±0.06 |  | 1.00±0.12 | 0.32±0.06 | 0.66±0.18 | 0.55±0.05 |
| TaIAA3-D | 1.00±0.12 | 0.54±0.13 | 0.31±0.12 | 0.29±0.07 |  | 1.04±0.02 | 0.33±0.05 | 0.52±0.11 | 0.30±0.15 |
| TaIAA4-B | 1.01± 0.20 | 0.55±0.10 | 0.41±0.09 | 0.48±0.06 |  | 1.01±0.04 | 0.42±0.16 | 0.68±0.08 | 0.39±0.03 |
| TaIAA4-D | 0.99±0.01 | 0.78±0.03 | 0.59±0.04 | 0.65±0.10 |  | 1.00±0.21 | 0.12±0.05 | 0.13±0.08 | 0.63±0.23 |
| TaIAA10-A | 1.00±0.16 | 0.91±0.17 | 0.82±0.08 | 1.16±0.17 |  | 1.00±0.06 | 0.16±0.04 | 0.23±0.06 | 0.91±0.09 |
| TaIAA11-B | 1.01±0.13 | 0.52±0.13 | 0.91±0.26 | 0.67±0.11 |  | 1.01±0.12 | 0.18±0.00 | 0.65±0.01 | 0.94±0.18 |
| TaIAA11-D | 1.10±0.06 | 0.91±0.11 | 0.66±0.03 | 0.75±0.12 |  | 1.01±0.10 | 0.88±0.40 | 0.79±0.04 | 0.67±0.22 |
| TaIAA13-D | 1.00±0.10 | 0.67±0.15 | 0.56±0.06 | 0.53±0.08 |  | 1.01±0.35 | 0.84±0.22 | 0.71±0.10 | 0.55±0.12 |
| TaIAA14-D | 1.00±0.33 | 0.63±0.14 | 0.42±0.01 | 1.21±0.18 |  | 1.00±0.04 | 0.08±0.13 | 0.24±0.07 | 0.41±0.07 |
| TaIAA18-A | 1.01±0.01 | 0.28± 0.02 | 0.38±0.08 | 0.61±0.07 |  | 1.02±0.31 | 0.08±0.07 | 0.12±0.06 | 0.41±0.00 |
| TaIAA19-A | 1.00±0.02 | 0.20±0.04 | 0.36±0.02 | 0.75±0.04 |  | 1.00±0.15 | 0.07±0.07 | 0.15±0.06 | 0.37±0.00 |
| TaIAA19-B | 1.00±0.13 | 0.19±0.00 | 0.30±0.00 | 0.39±0.07 |  | 1.02±0.03 | 0.06±0.03 | 0.08±0.01 | 0.38±0.01 |
| TaIAA19-D | 1.00±0.13 | 0.64±0.13 | 0.25±0.06 | 0.43±0.18 |  | 1.02±0.02 | 0.05±0.10 | 0.09±0.03 | 0.29±0.09 |
| TaIAA20-D | 1.01±0.00 | 0.29±0.00 | 0.10±0.01 | 0.83±0.05 |  | 1.00±0.14 | 0.02±0.00 | 0.17±0.04 | 0.16±0.07 |
| TaIAA21-B | 1.00±0.01 | 0.58±0.02 | 0.31±0.03 | 0.68±0.02 |  | 1.00±0.16 | 0.06±0.03 | 0.14±0.03 | 0.32±0.12 |
| TaIAA21-D | 1.00±0.03 | 0.68±0.03 | 0.27±0.05 | 0.69±0.17 |  | 1.00±0.13 | 0.05±0.05 | 0.14±0.03 | 0.29±0.04 |
| TaIAA22-A | 1.01±0.01 | 0.76±0.01 | 0.28±0.05 | 0.61±0.04 |  | 1.02±0.05 | 0.06±0.08 | 0.12±0.06 | 0.28±0.06 |
| TaIAA23-A | 1.00±0.01 | 0.73±0.02 | 0.18±0.08 | 0.42±0.01 |  | 1.00±0.27 | 0.04±0.00 | 0.08±0.01 | 0.17±0.09 |
| TaIAA25-A | 1.01±0.01 | 1.11±0.01 | 0.80±0.01 | 1.27±0.13 |  | 1.00±0.02 | 0.16±0.08 | 0.25±0.02 | 0.96±0.10 |
| TaIAA26-B | 1.00±0.25 | 1.32±0.12 | 1.05±0.29 | 4.62±0.06 |  | 1.00±0.24 | 0.21±0.06 | 0.92±0.02 | 1.29±0.05 |
| TaIAA26-D | 1.00±0.05 | 1.30±0.13 | 1.46±0.27 | 4.76±0.02 |  | 1.01±0.18 | 0.29±0.08 | 0.95±0.06 | 1.36±0.15 |
| TaIAA27-D | 1.00±0.02 | 1.18±0.05 | 1.92±0.30 | 5.47±0.04 |  | 1.01±0.14 | 0.38±0.13 | 1.09±0.33 | 1.87±0.37 |
| TaIAA31-A | 1.00±0.19 | 0.88±0.05 | 0.27±0.08 | 0.69±0.02 |  | 1.00±0.05 | 0.05±0.02 | 0.14±0.05 | 0.31±0.01 |
| TaIAA32-D | 1.07±0.03 | 0.81±0.03 | 0.69±0.05 | 0.90±0.11 |  | 1.00±0.02 | 0.14±0.06 | 0.18±0.06 | 0.68±0.04 |

Four-day-old wheat seedlings were exposed to 0.5 mM CaCl_2_ solution (pH 4.5) containing 0 (CK), 25, 50 or 100 μM AlCl_3_ for 24 h (A). Four-day-old wheat seedlings were exposed to 0.5 mM CaCl_2_ solution (pH 4.5) containing 0 or 50 μM AlCl_3_ for 0 (CK), 6, 12 and 24 h (B). Data are mean ± SD (n=3).
